# Supplementary material for: Physical limitations on broadband invisibility based on fast-light media
Source: Nat Commun. 2021 May 24;12:3041. doi: 10.1038/s41467-021-22972-w (PMC8144568; doi:10.1038/s41467-021-22972-w)
Supplement: Supplementary file 2 — Description of Additional Supplementary Files [file 41467_2021_22972_MOESM2_ESM.docx]

**Supplementary Movie 1:** Invisibility cloak made of fast-light media: Unstable temporal response

**Caption:** Left: Scattering poles in the complex frequency plane for an active cloak with parameters as in Fig. 2(c,d) of the main text. Right: Corresponding time-domain animation for broadband pulse propagation. The active cloak is clearly unstable as evidenced by the presence of unstable scattering poles and unbounded oscillations in its temporal response.
